# Evaluation of the Diagnostic Potential of uPAR as a Biomarker in Renal Biopsies of Patients with FSGS

**DOI:** 10.1155/2019/1070495

**Published:** 2019-05-02

**Authors:** Crislaine Aparecida da Silva, Liliane Silvano Araújo, Maria Luíza Gonçalves dos Reis Monteiro, Lívia Helena de Morais Pereira, Marcos Vinícius da Silva, Lúcio Roberto Cançado Castellano, Rosana Rosa Miranda Corrêa, Marlene Antônia dos Reis, Juliana Reis Machado

**Affiliations:** ^1^Discipline of General Pathology, Institute of Biological and Natural Sciences of Federal University of Triângulo Mineiro, Praça Manoel Terra, 330, Nossa Senhora da Abadia, 38025-015 Uberaba, Minas Gerais, Brazil; ^2^Discipline of Parasitology, Institute of Biological and Natural Sciences of Federal University of Triângulo Mineiro, Av. Getúlio Guaritá, No. 130, Nossa Senhora da Abadia, 38025-440 Uberaba, Minas Gerais, Brazil; ^3^Human Immunology Research and Education Group, Technical School of Health of Federal University of Paraíba, Cidade Universitária, 58059-900 João Pessoa, Paraíba, Brazil

## Abstract

Minimal change disease (MCD) and focal segmental glomerulosclerosis (FSGS) are primary glomerulopathies leading to proteinuria, known as podocytopathies, which share syndromic and morphological similarities. Morphological similarity occurs in cases of FSGS in which the sclerotic lesion was not sampled in renal biopsy, due to the focal nature of the disease. Differentiating these entities is very important, especially in cases of suspected FSGS but with sclerotic lesion not sampled, as they are diseases that apparently have different pathogenic mechanisms and prognosis. The difference in uPAR expression in situ among these two entities may be related to a distinct molecular mechanism involved in pathogenesis. Thus, finding biomarkers involved in the pathogenesis and that can also help in differential diagnosis is very relevant. The aim of this work was to evaluate the potential of urokinase-type plasminogen activator receptor (uPAR) as a biomarker in renal biopsies of patients with podocytopathies (*n* = 38). Immunohistochemistry showed that FSGS (*n* = 22) had increased uPAR expression in podocytes compared with both the MCD group (*n* = 16; *p* = 0.0368) and control group (*n* = 21; *p* = 0.0076). ROC curve (*p* = 0.008) showed that this biomarker has 80.95% of specificity in biopsies of patients with FSGS. Therefore, uPAR presented a high specificity in cases of podocytopathies associated with sclerosis and it can be considered a potential biomarker for FSGS.

## 1. Introduction

Glomerular diseases are among the leading causes of end-stage renal disease worldwide. The main clinical feature of patients with glomerulopathies is nephrotic syndrome (NS), which is characterized by nephrotic range proteinuria (>3.5 g/day), hypoalbuminemia (serum albumin < 3 g/dl), hyperlipidemia (serum cholesterol > 200 mg/dl), and edema, affecting both adults and children [1]. Podocytes are highly specialized epithelial cells with a unique architecture that covers the outer surfaces of glomerular capillaries, supporting the glomerular filtration barrier [2, 3]. Podocyte injury may lead to effacement of their extensions, the foot process, leading to proteinuria [4].

Focal segmental glomerulosclerosis (FSGS) and minimal change disease (MCD) are podocytopathies, characterized primarily by changes in podocytes [1] and have clinical and morphological similarities, sometimes making it difficult to distinguish between them. Morphological similarity occurs specially in cases of nonsampled FSGS in renal biopsy, as sclerosis in this disease, by definition, is a focal finding: not all glomeruli are affected [5]. Thus, it is very important to find biomarkers involved in pathogenesis of these entities and that, in addition, can help in diagnosis [6, 7].

Some authors distinguish these entities based on differences in their clinical presentations and histological characteristics [8, 9], as opposed to others who believe they are different manifestations of the same progressive disease, in which FSGS would be an advanced stage [10]. Pathogenesis of these entities is controversial, but it seems to be related to structural and/or molecular podocyte changes, and some proteins have also been associated with renal damage and proteinuria [1]. In this way, uPAR/suPAR has been proposed to have a role in FSGS pathogenesis [11–15].

uPAR is a membrane-bound 45-55 kDa protein with three domains (DI, DII, and DIII) linked to glycosylphosphatidylinositol (GPI). It is found in several immunologically active cells, as well as in podocytes [16]. Once bound to its ligand, it can promote cell adhesion, migration, and cell proliferation disorders [17]. In podocytes, it was observed that uPAR is able to activate *α*v*β*3 integrin promoting cell mobility and activation of small GTPases, such as Cdc42 and Rac1, thus allowing contraction of podocytes, which acquire motility and consequently foot process effacement, as well as development of proteinuria [18].

uPAR is released from cell surface in a soluble form, suPAR, which can be found in several body fluids, including plasma, urine, saliva, and cerebrospinal fluid at different levels and with similar functions to uPAR [16].

Due to the common recurrence of FSGS after transplantation, it is possible that circulating factors may be involved in the pathogenesis of this disease [19]. suPAR was thought to be this possible circulating factor, as circulating suPAR could activate *α*v*β*3 integrin similarly to membrane-bound uPAR in podocytes [11].

There are literature controversies concerning the role of uPAR/suPAR as a biomarker of FSGS as suPAR levels are also increased in other glomerular diseases [20–23]. However, a growing body of evidence suggests a role of suPAR as a scaring factor in FSGS [11, 12, 19, 24, 25].

suPAR has been proposed to have a role in FSGS pathogenesis although this is debated and unclear. Despite this, there is no study evaluating the potential of uPAR staining in renal biopsy as a way of differentiating FSGS from MCD. So, we decided to explore the role of uPAR in differentiating FSGS and MCD.

## 2. Methods

### 2.1. Patients

Thirty-eight cases of podocytopathies were selected, comprising patients with FSGS (*n* = 22) and MCD (*n* = 16) from the Nephropathology Service of General Pathology Discipline of Federal University of Triângulo Mineiro (UFTM), Uberaba, Minas Gerais State, Brazil. The groups were divided as follows: (a) the FSGS group, defined by the presence of segmental sclerosis (increase in mesangial matrix) and, in electron microscopy, foot process effacement, and (b) the MCD group, defined by foot process effacement as an isolated finding in electron microscopy. The control group (*n* = 21) was composed of autopsy kidneys from patients whose death was not related to renal or infectious diseases.

The ethics and research committee of Federal University of Triângulo Mineiro approved this study with the number 1.715.838.

### 2.2. Renal Histopathology

Renal specimens were evaluated by direct immunofluorescence, light, and electron microscopy similar to the Huang et al. technique [26]. For direct immunofluorescence, immunoglobulins IgG, IgM, and IgA; kappa and lambda light chains; complement fractions C3 and C1q; and fibrinogen were detected by fluorescein isothiocyanate- (FITC-) conjugated antibodies (Dako, Copenhagen, Denmark) on frozen tissues. For light microscopy, paraffin sections were stained with hematoxylin and eosin, sirius red, silver methenamine stain (PAMS), and Masson's trichrome. For electron microscopy, in brief, tissue was fixed in 2.5% Karnovsky +0.2% ruthenium red and latter fixed in osmium tetroxide 2% and then dehydrated in graded alcohols and acetone solutions and embedded in Epon 812. Ultrathin sections were cut with 60 nm thickness and placed on nickel grids. Then, ultrathin sections were stained with uranyl acetate and examined with a transmission electron microscope EM-900 (Zeiss, Germany).

### 2.3. Immunohistochemistry for uPAR

Renal biopsy sections were fixed in paraformaldehyde for immunohistochemistry, and peroxidase and protein blockage was done using Novolink blocker for 50 minutes each. Then, human anti-*uPAR* antibody (1 : 50) was incubated overnight at 4°C. After, slides were incubated with Post Primary (Novolink Polymer Detection System Kit, BL, UK) for 50 minutes at room temperature and then incubated with the polymer (Novolink Polymer Detection System Kit, BL, UK) for 50 minutes. The material was then allowed to react with DAB substrate for staining (1,4-dideoxy-1,4-imino-D-arabinitol-diaminobenzidine) (Liquid DAB, Dako, Carpinteria, CA, USA) for 2 minutes, and sections were counterstained with hematoxylin and analyzed using a light microscope.

### 2.4. uPAR Immunostaining Quantification

Immunostained cells in glomeruli were counted as uPAR-positive cells, in order to obtain its density in a glomerular area. The result was expressed in cell density (cell/mm^2^), in a technique adapted from Venkatareddy et al. [27].

### 2.5. Statistical Analysis

Statistical analysis was performed with the program GraphPad Prism version 6.0. Normality was tested by the Shapiro-Wilk test. For comparison analysis, the Kruskal-Wallis test (*H*) was used followed by the Dunn posttest. In contingency table analysis, Fisher's exact test was used. uPAR diagnostic performance in renal biopsy was evaluated with the receiver operating characteristic curve (ROC curve) using sensitivity, specificity, and area under the curve (AUC) with 95% of confidence intervals (CI); cutoff points were calculated using nonparametric methods. Differences were considered statistically significant when *p* < 0.05.

## 3. Results

Patients' median age was 35.5 years, ranging from 15 to 70 years. Twenty were men and 18 were women. Patients with FSGS presented a more unfavorable clinical profile, with higher levels of creatinine (*p* = 0.0156; *U* = 56.50), of proteinuria (*p* = 0.0234; *U* = 102.0), and increased prevalence of hypertension (Fisher's exact test *p* = 0.0009). Patients' profile is detailed in Table 1.

**Figure 1:**
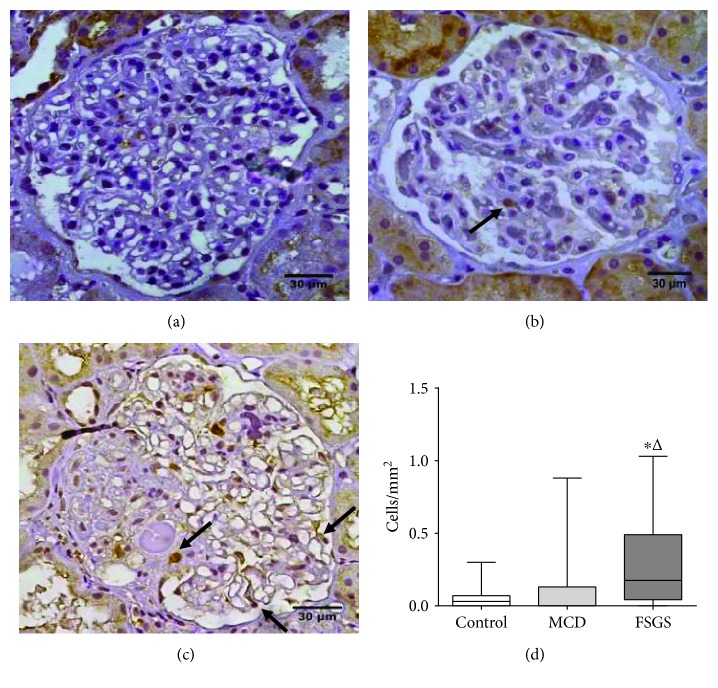
Evaluation of uPAR in podocytopathies and in the control group. uPAR immunolabeling evidenced by arrows (a) in the control group; (b) in a case of MCD, in which only rare cells are labeled; and (c) in a case of FSGS in which there are diffusely marked cells, including cells still viable in the sclerotic segment. (d) uPAR glomerular expression. Kruskal-Wallis one-way analysis of variance followed by Dunn's multiple comparison. Horizontal lines represent the median, bars represent 25-75% percentiles, and vertical lines represent 10-90% percentiles. MCD: minimal change disease; FSGS: focal segmental glomerulosclerosis; ∗: significant differences between the FSGS versus the control group; Δ: significant differences between the FSGS versus the MCD group.

A previous study has shown that suPAR may be related to FSGS pathogenesis. So, we hypothesized that patients with FSGS have greater in situ uPAR expression in glomeruli than patients with MCD. It was observed that uPAR podocyte expression was increased in the FSGS group (Figure 1(c)) compared to both the control group (Figure 1(a); *p* = 0.0076; Figure 1(d)) and MCD group (Figure 1(b); *p* = 0.0368; Figure 1(d)). The expression of uPAR was diffuse in glomeruli without sclerosis and in viable glomerulus cells within segmental sclerosis.

**Figure 2:**
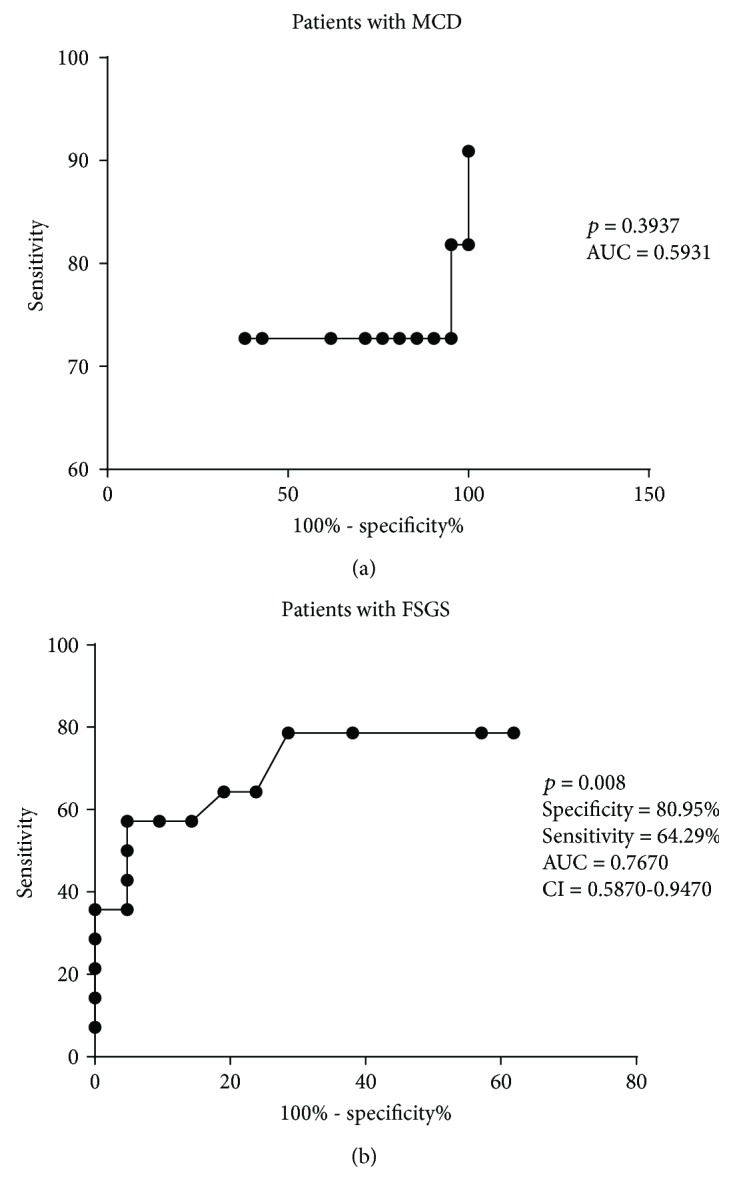
Receiver operating characteristic (ROC) curve to evaluate the potential diagnosis of uPAR. (a) ROC curve showed that uPAR has no potential diagnosis in patients with MCD. (b) ROC curve showed that uPAR can be considered a potential biomarker for FSGS. AUC: area under the curve; CI: confidence interval.

As uPAR expression was increased in biopsies of patients with FSGS, we sought to examine how useful uPAR immunohistochemistry staining would be to FSGS diagnosis. Using a ROC curve, an optimum cutoff point at 0.08 cells/mm^2^ labeled with uPAR was found to have 64.29% of sensitivity, 80.95% of specificity, and AUC of 0.7670 (95% CI of 0.5870-0.9470, *p* = 0.008, Figure 2(b)). The same was not observed when the ROC curve was used to evaluate potential diagnosis of uPAR in MCD (*p* = 0.3937, Figure 2(a)).

## 4. Discussion

FSGS and MCD are common glomerular diseases which present clinical similarities as proteinuria and/or nephrotic syndrome but have different clinical evolution. In this study, patients with FSGS presented higher serum levels of creatinine and presence of arterial hypertension, which is consistent with literature, as this entity presents an unfavorable clinical course, does not respond well to corticosteroids, and progresses to renal failure in a variable period of time [28]. About 25 to 50% of patients with FSGS have decreased renal function, and arterial hypertension is present in about 60% of them [29].

In addition to clinical similarities, both entities present morphological similarities as foot process effacement, and, in cases in which FSGS sclerosis is not sampled, the differential diagnosis between these two diseases becomes challenging.

In this scenario, we looked for a possible biomarker that would help differentiate these two entities. We chose uPAR/suPAR, which has been proposed to have a role in FSGS pathogenesis [11–15].

Of note, our MDC patients had low levels of proteinuria, which is not in line with literature [29]. However, these patients presented important hypoalbuminemia, characterizing the nephrotic condition. This hypoalbuminemia reflects low levels of serum protein, which results in less protein in urine. Although there is a consensus recommendation for renal biopsy only in patients with nephrotic range proteinuria, renal biopsy indications differ considerably among nephrologists [30]. Thus, it is possible that the presence of hypoalbuminemia in our patients contributed to the indication of biopsy even with nonnephrotic proteinuria.

In this study, we demonstrate that patients with FSGS have increased uPAR expression in renal biopsy compared to patients without renal alteration and patients with MCD.

The protein uPAR is expressed in human glomerular cells and one of them is the podocyte, as verified by double immunofluorescence labeling with synaptopodin, a podocyte marker. The same was observed in glomeruli of animal models, where uPAR expression in all models of proteinuria was substantially increased in glomerular cells, including podocytes. By analyzing vitronectin expression, a protein that binds to uPAR, a labeling pattern like that of uPAR was observed in human and animal podocytes. In addition, culture of podocytes treated with puromycin aminonucleoside (PAN) and LPS revealed increased uPAR expression in these cells with labeling preferentially located on cell membrane [18].

Using an animal model knockout for PLAUR, uPAR has been shown to play a direct role in the regulation of podocyte structure and function, as uPAR deficiency protects against LPS-induced proteinuria and podocyte injury [18].

A possible mechanism for foot process effacement is through integrin activation as uPAR is a GPI-anchored protein without a cytoplasmic tail and uPAR signal transduction seems to be through lateral interactions with membrane proteins such as integrins [31].

After identification of an integrin-interacting sequence located in domain 2 of uPAR that activates the *α*v*β*3 signal-dependent signaling pathways [32], it was observed through immunogold that the location of *α*v*β*3 integrin and uPAR was similar in podocytes, suggesting uPAR interacts with this integrin. In addition, using an antibody that inhibits *β*3 integrin function, mice did not develop proteinuria in response to LPS [31]. Another evidence of uPAR interaction with *α*v*β*3 integrin comes from an experiment with animal knockout for urokinase, the major ligand of uPAR, in which, after treatment with LPS, animals presented proteinuria, showing podocyte lesion triggered by uPAR is independent of its ligand [18].

It is believed that both urinary and serum suPAR can activate *β*3 integrin in a similar manner to the binding of uPAR to podocyte membrane. To study human podocytes *β*3 integrin activity, AP5, an epitope-recognizing antibody was used and a strong AP5 labeling was observed along cell membrane of podocytes incubated with urine of FSGS patients. This expression was reduced with the addition, in the incubation, of an antibody blocking uPAR [26]. The same was observed in podocytes cultured with plasma of patients with recurrent FSGS [11].

The role of uPAR/suPAR as a FSGS biomarker is still controversial in literature. However, in this study involving podocytopathies, we observed that uPAR has a specificity for FSGS and can be considered a scaring factor in this disease.

### 4.1. Limitation of Study

Although our sample came from several Brazilian regions, it would be advisable that studies involving human samples would be replicated in different cohorts and ethnicities.

## 5. Conclusions

Our results demonstrated that uPAR has high specificity for FSGS cases. Therefore, this marker may be useful in the diagnosis of FSGS in renal biopsies in which FSGS is suspected, but the sclerotic lesion was not sampled.

## Figures and Tables

**Table 1:** Clinical-epidemiological profile of patients.

	MCD (*n* = 16)	FSGS (*n* = 22)	*p* value
Age			
Mean ± SD	37.37 ± 14.90	38.32 ± 14.92	0.8484
Median (min–max)	34.5 (15-70)	36 (16-73)	
Gender, *n* (%)			
Male	6 (37.5%)	14 (63.64%)	0.1881
Female	10 (62.5%)	8 (36.36%)	
Creatinine (mg/dl)			
Mean ± SD	0.97 ± 0.51	1.52 ± 0.65	0.0156^∗^
Median (min–max)	0.9 (0.5-2.4)	1.4 (0.8-3.0)	
Proteinuria (g/24 h)			
Mean ± SD	2.83 ± 2.15	5.13 ± 3.37	0.0234^∗^
Median (min–max)	2.15 (0.16-6.19)	4.35 (1.24-14.46)	
Albumin (mg/dl)			
Mean ± SD	2.85 ± 1.01	2.72 ± 1.01	0.8258
Median (min–max)	2.6 (1.4-4.6)	3.3 (0.8-3.9)	
Hematuria			
Yes	8 (50.00%)	6 (27.27%)	0.4905
No	8 (50.00%)	11 (50.00%)	
Hypertension			
Yes	4 (25.00%)	16 (72.73%)	0.0009^∗^
No	8 (50.00%)	1 (4.54%)	

^∗^ *p* < 0.05.

## Data Availability

The data used to support the findings of this study are included within the article.
